# Supplementary material for: Association between resting-state functional brain connectivity and gene expression is altered in autism spectrum disorder
Source: Nat Commun. 2022 Jun 9;13:3328. doi: 10.1038/s41467-022-31053-5 (PMC9184501; doi:10.1038/s41467-022-31053-5)
Supplement: Supplementary file 1 — Supplementary Information [file 41467_2022_31053_MOESM1_ESM.pdf]

Association between resting-state functional brain connectivity and gene expression is altered in autism spectrum disorder

Stefano Berto[1], Alex Treacher[2], Emre Caglayan[1], Danni Luo[2], Jillian R. Haney[3,4,5], Michael J. Gandal[3,4,5,6], Daniel H. Geschwind[3,4,5,6], Albert Montillo[2,7,8] and Genevieve Konopka[1]

[1] Department of Neuroscience, UT Southwestern Medical Center, Dallas, TX 75390, USA.
[2] Lyda Hill Department of Bioinformatics, UT Southwestern Medical Center, Dallas, TX 75390, USA.
[3] Program in Neurobehavioral Genetics, Semel Institute, David Geffen School of Medicine, University of California, Los Angeles, Los Angeles, CA 90095, USA.
[4] Department of Neurology, Center for Autism Research and Treatment, Semel Institute, David Geffen School of Medicine, University of California, Los Angeles, Los Angeles, CA 90095, USA.
[5] Department of Psychiatry, Semel Institute, David Geffen School of Medicine, University of California, Los Angeles, Los Angeles, CA 90095, USA.
[6] Department of Human Genetics, David Geffen School of Medicine, University of California, Los Angeles, Los Angeles, CA 90095, USA.
[7] Department of Radiology, University of Texas Southwestern Medical Center, Texas, USA
[8] Advanced Imaging Research Center, University of Texas Southwestern Medical Center, Texas, USA

Supplementary Figures 1-8

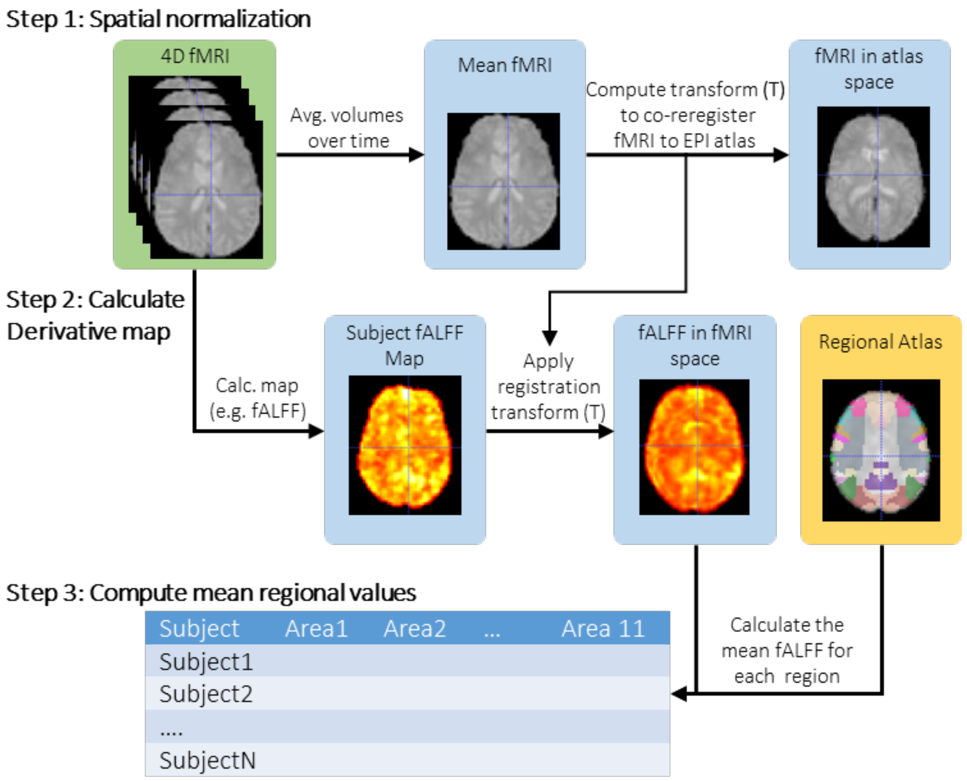

Supplementary Figure 1: Overview of image analysis pipeline. Three steps are used to calculate regional values from MRI. Step 1: Subject fMRI (green) is spatially normalized to atlas space. Step 2: Local functional activity measures are derived for each subject (e.g. fALFF) and coregistered to atlas space. Step 3: Mean regional measures are computed using the atlas (yellow) for each region for each subject.

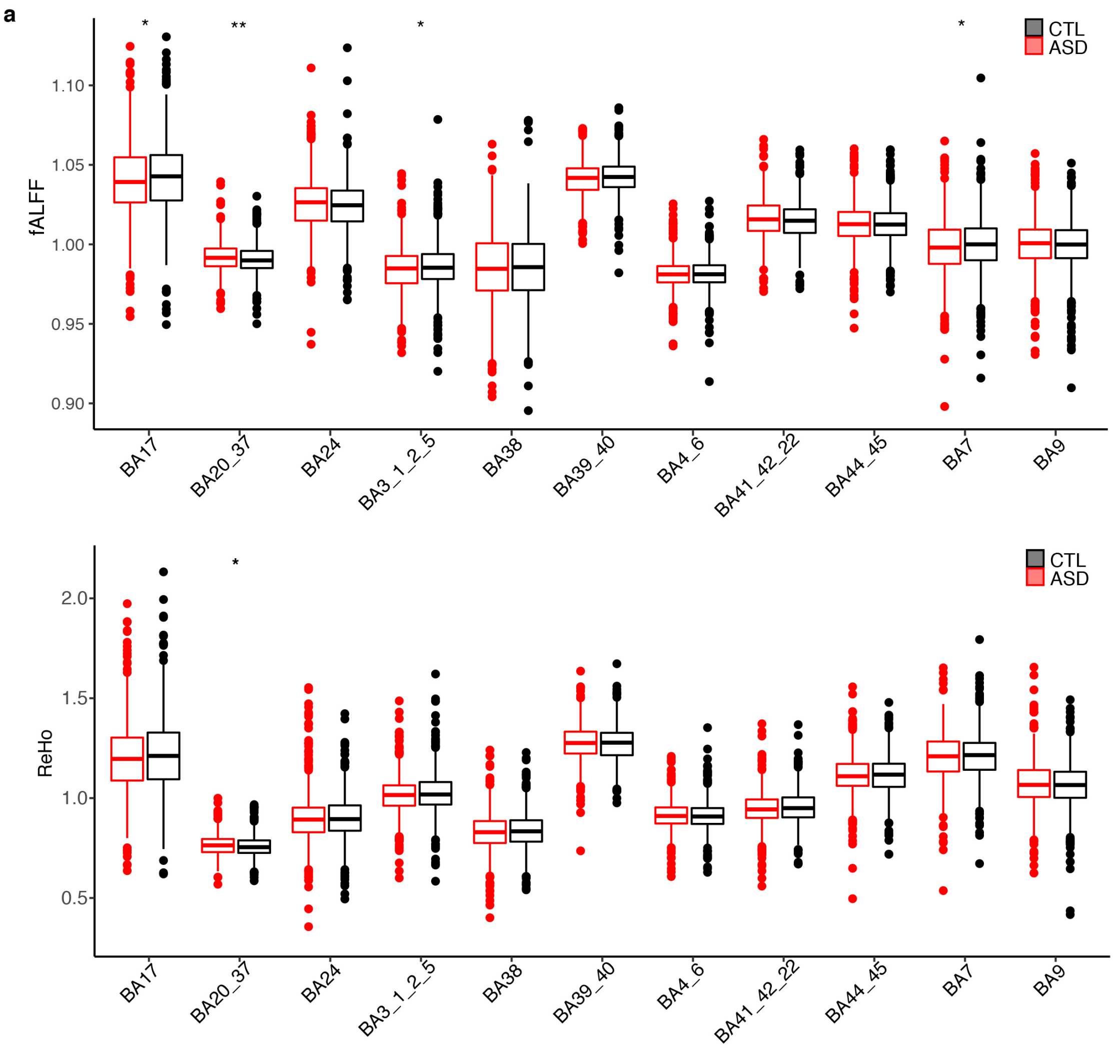

Supplementary Figure 2. rs-fMRI data quality control. a, Boxplots of fALFF and ReHo measurements comparison between ASD (red) and CTL (black) across multiple ROIs analyzed. Stars correspond to the significant differences between ASD and CTL based on one-sided Wilcoxon rank sum's test (** p = 0.001, * p = 0.05). Boxes extend from the 25th to the 75th percentiles, the center lines represent the median. ASD: N = 606 biologically independent samples, CTL: N = 710 biologically independent samples. Exact P-value fALFF: BA17 p = 0.048, BA20_37 p = 0.001, BA7 p = 0.04. Exact P-value ReHo: BA20_37 p = 0.026.

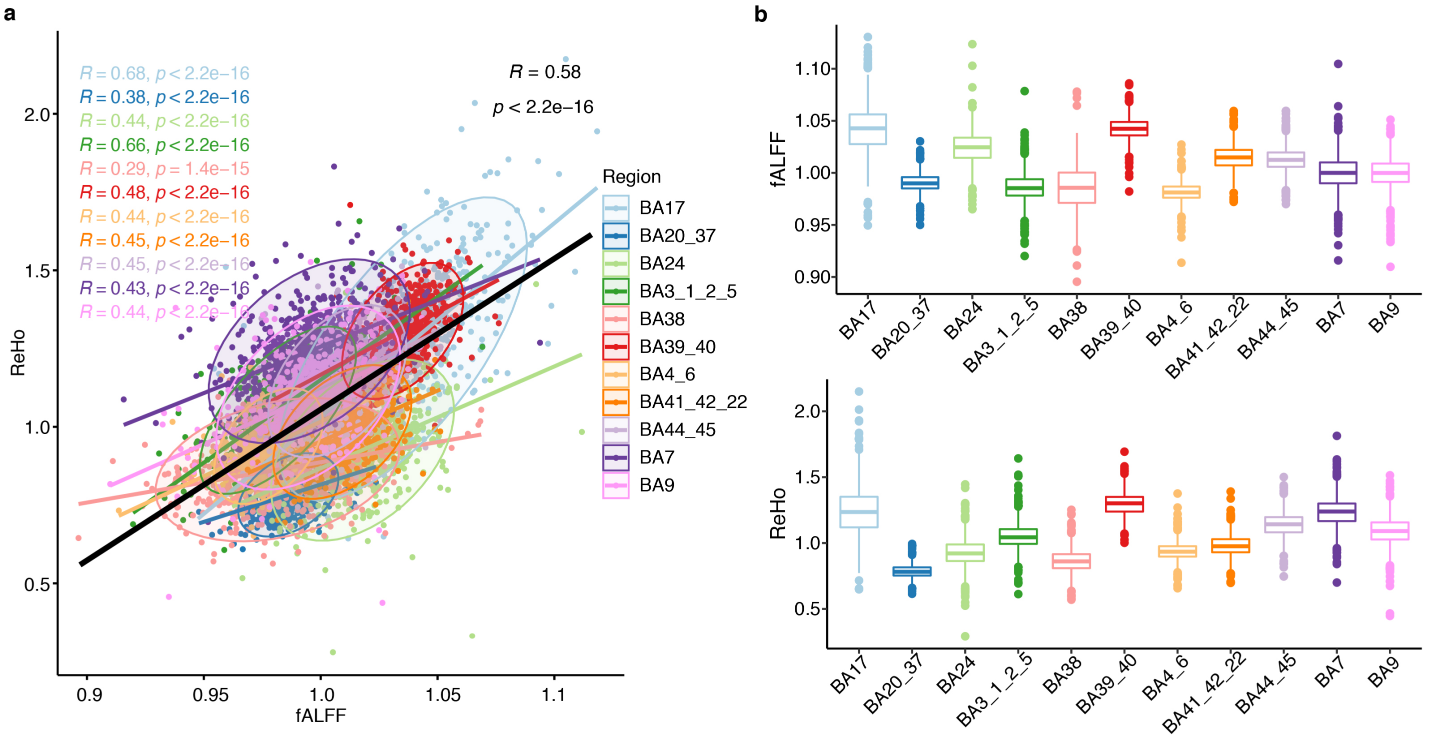

Supplementary Figure 3. Comparisons of the two types of rs-fMRI measurements. a, Scatter plot comparing fALFF (X-axis) and ReHo (Y-axis) between the 11 ROIs analyzed in CTL. Spearman rank *rho* values and associated p-values are shown colored by ROIs. Black line corresponds to the across-ROIs (pancortical) correlation (Spearman's rank correlation test, two-tailed). **b,** Distribution of fALFF and ReHo measurements in the 11 ROIs analyzed in CTL. Boxes extend from the 25th to the 75th percentiles, the center lines represent the median. ASD: N = 606 biologically independent samples, CTL: N = 710 biologically independent samples.

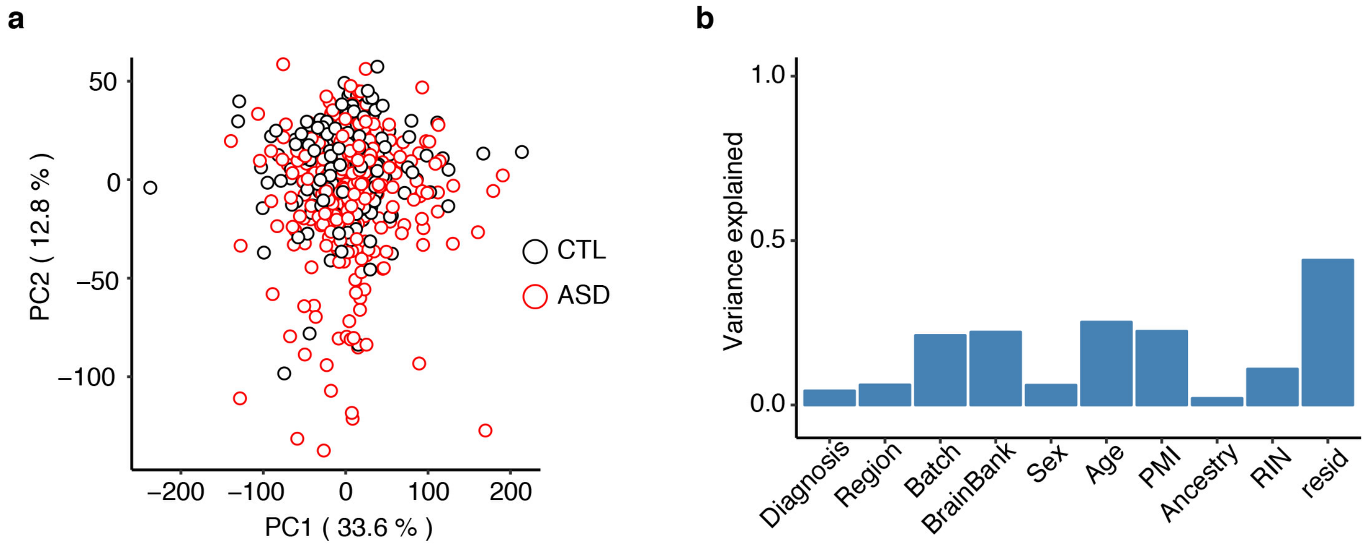

Supplementary Figure 4. **RNA-seq data quality control and covariate metrics. a**, Principal component analysis based on the RNA-seq data of all the subjects used in this study. **b**, Variance explained by each covariate adjusted across 10 principal components.

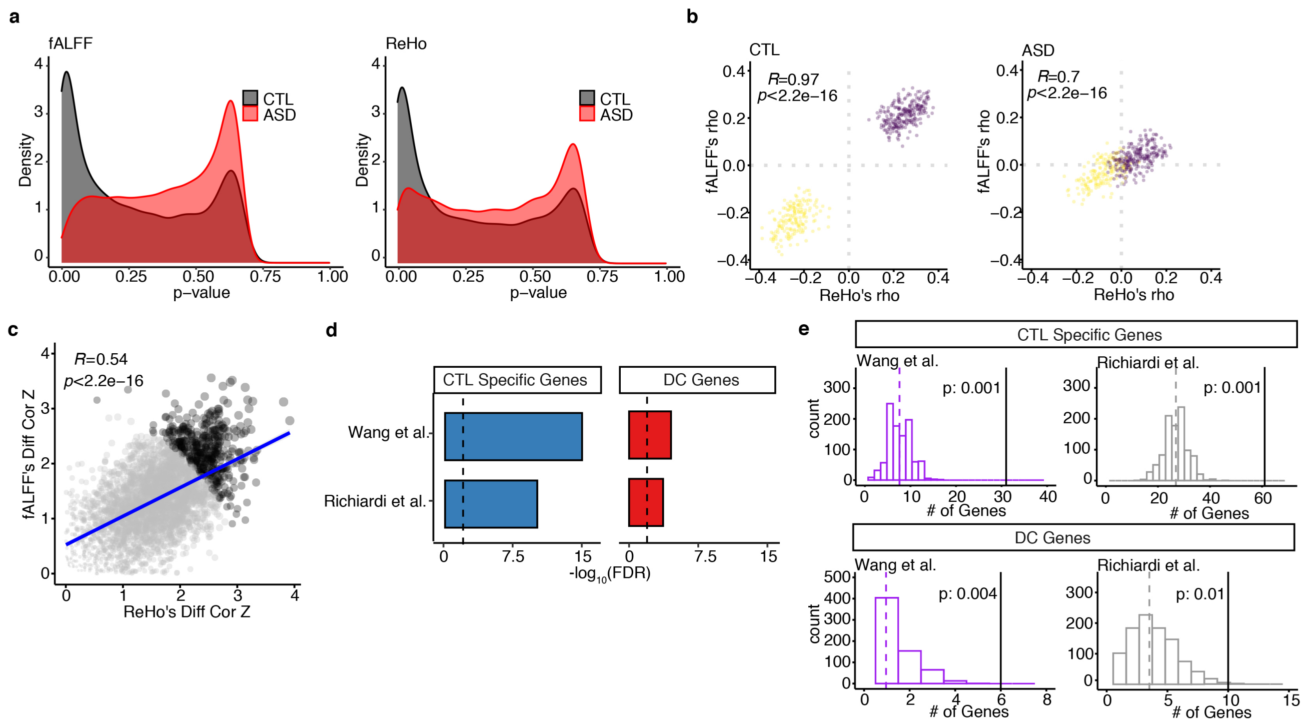

Supplementary Figure 5. Comparison of differentially correlated genes using either imaging metric or with other published datasets. a, Distribution of Spearman's rank p-values in both rs-fMRI measurements for ASD and CTL. **b**, Scatterplot depicting the correlation between CTL and ASD fALFF and ReHo *rho* values for the differentially correlated genes (DC genes) (Spearman's rank correlation test, two-tailed). **c**, Scatterplot depicting the correlation between fALFF and ReHo differentially correlated effect sizes (Spearman's rank correlation test, two-tailed). **d**, Barplot depicting the -log$_{10}$(FDR) of the overlap between CTL specific and DC Genes with previously published rs-fMRI genes (Fisher's Exact Test). **e**, Permutation tests of the presented overlaps. Brain expressed genes were randomly permuted matching the number of rs-fMRI genes identified in the two independent studies. Histograms show the distribution of overlapped genes between permuted data and genes found in this study. Overlaps and p-values were obtained using 1000 random permutations. Black lines indicate the original overlap.

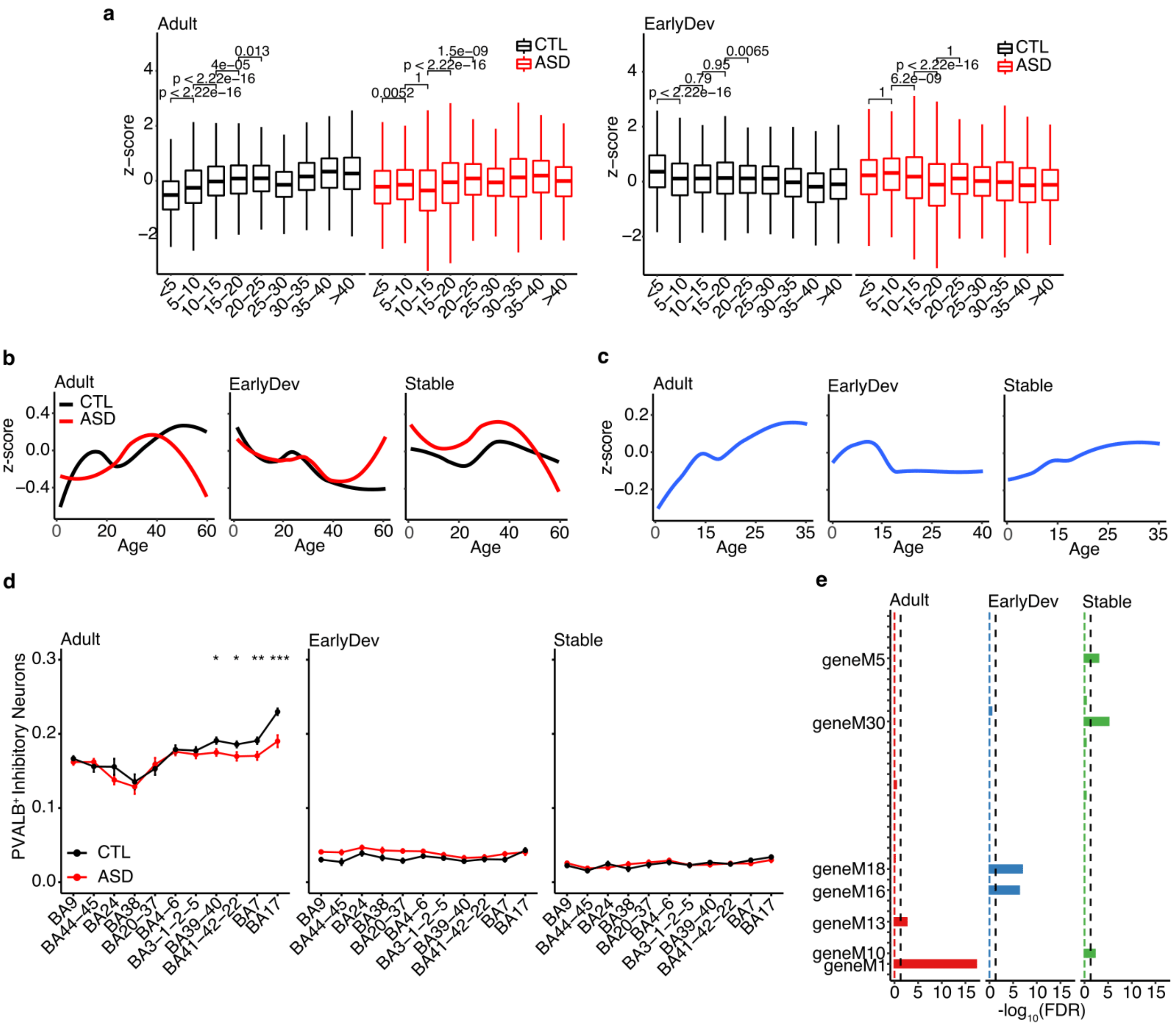

Supplementary Figure 6. **Gene expression comparisons of ASD and CTL across developmental trajectories. a**, Statistical comparison of developmental trajectories. Samples were divided into age brackets and age brackets were compared by one-sided t-test (alternative hypothesis: greater with increasing age in Adult, less with increasing age in EarlyDev). Numbers on graph are p-values, y-axis indicates Z-scored gene expression. Note that z-score spans a larger interval compared to Figure 4a. ASD: N = 360 biologically independent samples, CTL: N = 302 biologically independent samples. Boxes extend from the 25th to the 75th percentiles, the center lines represent the median. **b,** Developmental trajectories after equalizing diagnosis-region groups by random subsampling. Loess regression was used to fit smooth curve for the values of all genes per cluster across development. Smooth curves are shown with 95% confidence bands. Y-axis indicates Z-scored gene expression. **c**, Developmental trajectories of Adult, EarlyDev and Stable gene clusters using BrainSpan atlas (similar to Figure 4a). **d,** Line chart showing the median with standard error of *PVALB*+ interneurons imputed proportions across the 11 regions analyzed based on Adult, EarlyDev, Stable genes. Stars correspond to the significant differences between ASD and CTL based on one-sided Wilcoxon rank sum's test (** p = 0.01, * p = 0.05). Y-axis indicates cortical regions by anterior-to-posterior ordering. **e**, Barplot of enrichment between developmental clusters and modules associated with autism, bipolar disorder, and schizophrenia (Y-axis) from an independent study.

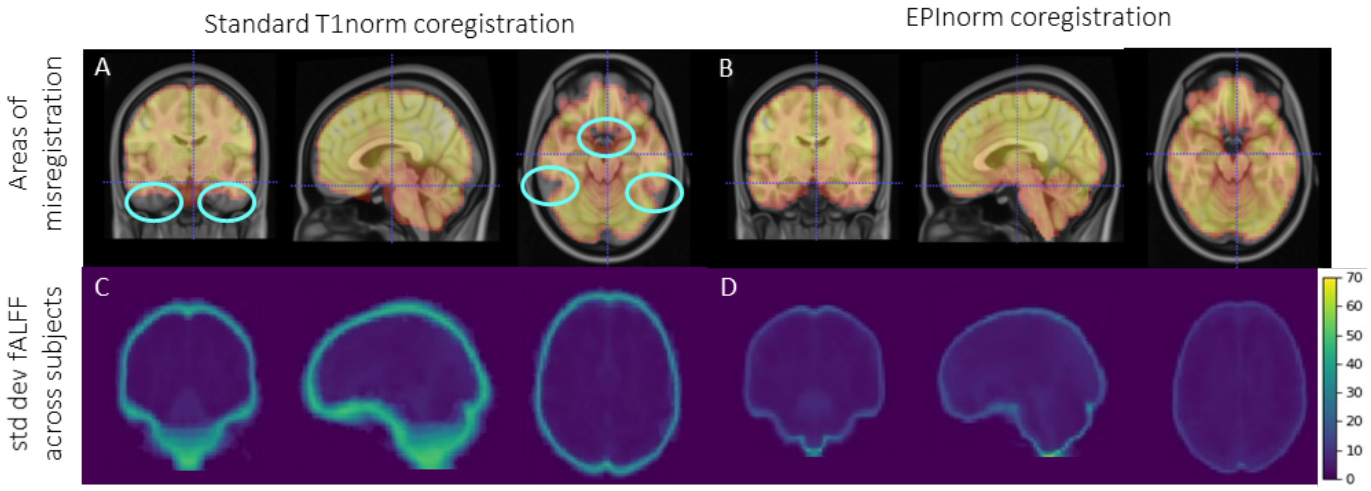

Supplementary Figure 7: **Quantitative comparison of EPInorm-based versus T1norm-based co-registration.** Each panel shows a mid-coronal image (left), mid-sagittal image (middle) and mid-axial image (right). Top panels show fMRI (yellow/red overlay) coregistered to MNI T1 anatomical atlas (underlay) using (A) T1norm-based co-registration and (B) EPI based registration. Both co-registrations are 3-dimensional. EPInorm based co-registration better aligns the derivative maps (fALFF and ReHo) to brain anatomy and reduces areas of misregistration (blue circles). Bottom panels show the standard deviation for fALFF values across subjects using (C) T1norm-based co-registration and (D) EPInorm-based co-registration.

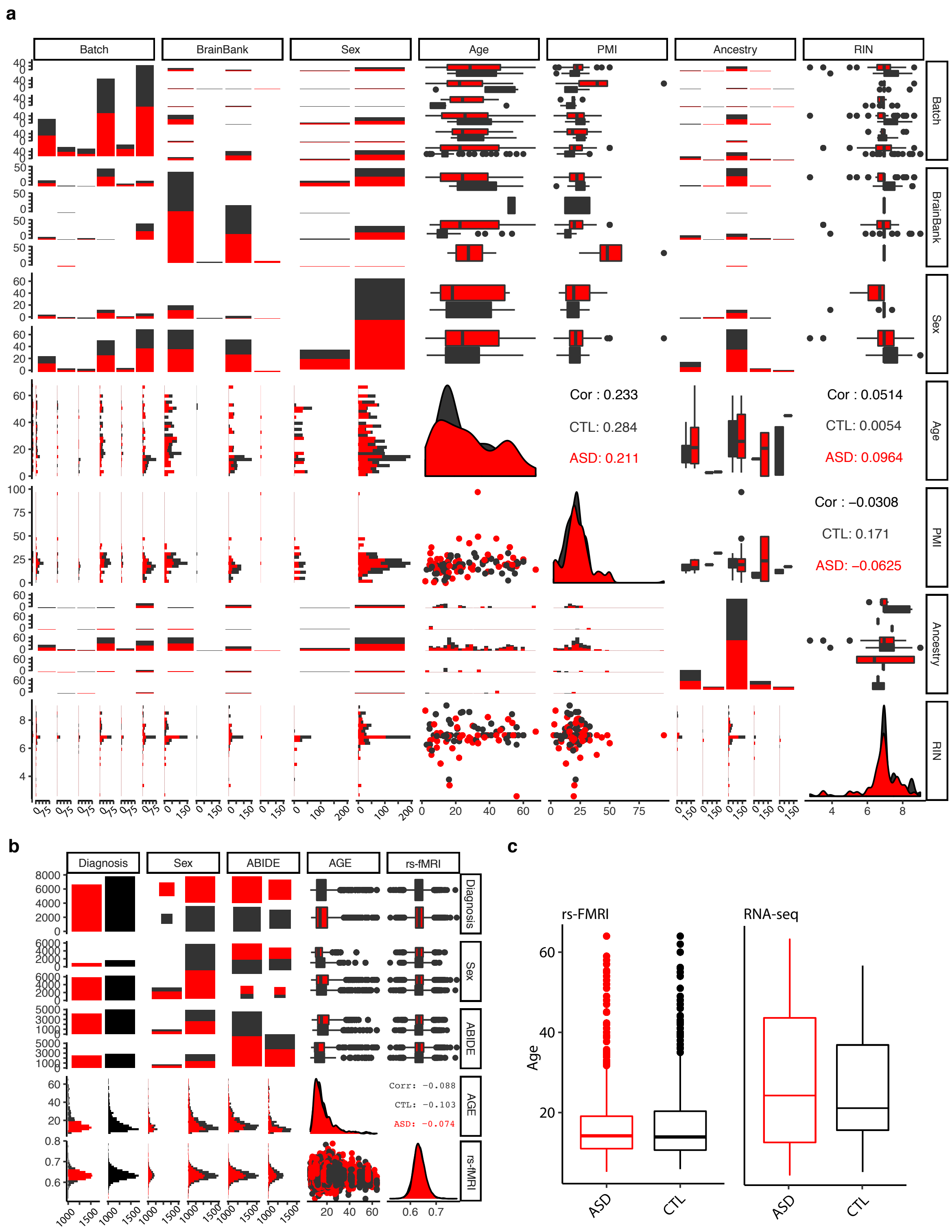

Supplementary Figure 8. Averaged demographic information for ASD and CTL groups. a) Pairwise comparison of demographic information containing biological and technical covariates for RNA-seq. In red: ASD subjects; in black: control. **b**) Pairwise comparison of demographic information containing biological and technical covariates for rs-fMRI. In red: ASD subjects; in black: control. **c**) Distribution of the age of the individuals who provided data for either RNA-seq or rs-fMRI studies. RNA-seq: ASD: N = 360 biologically independent samples, CTL: N = 302 biologically independent samples. rs-fMRI: ASD: N = 606 biologically independent samples, CTL: N = 710 biologically independent samples. Boxes extend from the 25th to the 75th percentiles, the center lines represent the median.
