# Supplementary material for: Association between resting-state functional brain connectivity and gene expression is altered in autism spectrum disorder
Source: Nat Commun. 2022 Jun 9;13:3328. doi: 10.1038/s41467-022-31053-5 (PMC9184501; doi:10.1038/s41467-022-31053-5)
Supplement: Supplementary file 3 — Description of Additional Supplementary Files [file 41467_2022_31053_MOESM3_ESM.pdf]

Description of Additional Supplementary Files

Supplementary Data 1. rs-fMRI values and statistics

Supplementary Data 2. DC genes database

Supplementary Data 3. Deconvolution Statistics

Supplementary Data 4. Leave One Region Out Statistics
